# Enhancing the electrical properties of graphite nanoflake through gamma-ray irradiation

**DOI:** 10.1038/s41598-022-19232-2

**Published:** 2022-09-01

**Authors:** Anh Tuan Nguyen, Youlim Lee, Phuong Quang Hoang Nguyen, Przemyslaw Dera, Sang-Hee Yoon, Woochul Lee

**Affiliations:** 1 Department of Mechanical Engineering, University of Hawaii at Manoa, Honolulu, HI 96822 USA; 2 Bioinspired Engineering Laboratory, Department of Mechanical Engineering, Inha University, Incheon, 22212 Republic of Korea; 3 Hawaii Institute of Geophysics and Planetology, University of Hawaii at Manoa, Honolulu, HI 96822 USA

**Keywords:** Nanoscience and technology, Graphene, Nanoscale materials, Engineering, Aerospace engineering, Electrical and electronic engineering, Mechanical engineering, Materials science, Materials for devices, Nanoscale materials

## Abstract

Understanding changes in material properties through external stimuli is critical to validating the expected performance of materials as well as engineering material properties in a controlled manner. Here, we investigate a change in the c-axis electrical properties of graphite nanoflakes (GnFs) induced by gamma-ray irradiation, using conductive probe atomic force microscopy (CP-AFM). The fundamentals behind the change in their electrical properties are elucidated by analyzing the interlayer spacing, graphitization, and morphology. An increase in gamma-ray irradiation dose for GnFs leads to an exponential increase in the electrical conductance and a gradual decrease in the interlayer spacing, while accompanying indistinguishable changes in their morphology. Our experimental results suggest that the c-axis electrical conductance enhancement of GnFs with gamma-ray irradiation might be attributed to a reduction in interlayer spacing, though the created defects may also play a role. This study demonstrates that gamma-ray irradiation can be a promising route to tailor the electrical properties of GnFs.

## Introduction

Graphite nanoflake (GnF), one of the advanced carbon-based materials, possesses extraordinary properties such as low density, high temperature/water resistance, remarkable lubricity, excellent flexibility, etc^1,2^. Owing to its excellent properties, GnF has emerged as a promising nanofiller for functional polymer matrix composites that are widely used in sensors^3^, fire retardants^4–6^, energy storage devices^7^, etc. The need for materials that can tolerate harsh environments sparked investigations of carbon-based materials^8^. Specifically, graphite^9^, graphene^10^, and carbon nanotubes^11^ have been extensively studied for applications in harsh radiation environments such as a satellite and spent nuclear fuel. For gamma-ray irradiation on carbon-based materials, most of the previous efforts with carbon-based materials concentrated on high gamma radiation doses of MGy or higher, and investigated mainly changes in the internal structures (e.g., structural order, graphitization, etc.)^12–15^. However, the effects of low gamma radiation doses of few kGy or lower on the structures and properties of carbon-based materials have rarely been reported. To the best of our knowledge, no study has been conducted on the GnF irradiated by gamma-ray and on a variation in the electrical conductance of GnF induced by gamma-ray irradiation. Recently, the versatility of GnF has led to extensive studies on polymer nanocomposites reinforced with GnF for radiation environments. For example, Kim et al. tried to monitor the structural integrity of dry storage canisters for spent nuclear fuels using GnF-based conductive polymer nanocomposites^16^. Although the nanocomposites are to be exposed to gamma-ray from radioactive waste, the study contains no information on the structural and property changes of GnF by gamma-ray irradiation. Here, we characterize a change in the electrical properties of the GnF caused by low-level gamma radiation doses and elucidate a primary cause of the change. Conductive probe atomic force microscopy (CP-AFM) is employed to measure the c-axis electrical conductance of a set of GnF samples exposed to different gamma radiation doses of 0.0 (no radiation) to 5.0 kGy. Next, a variety of characterization methods, including Raman spectroscopy, X-ray powder diffraction (XRD), scanning electron microscope (SEM), and transmission electron microscopy (TEM), are used to obtain the interlayer spacing, graphitization, and morphology of the GnF samples. Next, we correlate the change in electrical conductance with internal structural parameters of gamma-ray irradiated GnF to find a relation. The potential for enhancing and tailoring the electrical properties of GnF through gamma-ray irradiation is also addressed.

## Materials and methods

### Materials

Natural graphite was purchased from Asbury Carbons (NJ, USA). Potassium permanganate (KMnO_4_) and nitric acid (HNO_3_) were obtained from Sigma-Aldrich (MO, USA). All chemicals used for GnF synthesis were of reagent grade and used as received.

### Preparation of GnF

GnF samples were prepared from natural graphite using the method described in our previous study^1^. In brief, natural graphite was intercalated by mixing with KMnO_4_ (oxidant) and HNO_3_ (intercalant) at a weight ratio of 1:1:2 (graphite:KMnO_4_:HNO_3_), followed by microwave irradiation at 700 W for 60 s for graphite exfoliation^17^. After washing off with deionized (DI) water, the exfoliated graphite in DI water was fragmented using an ultrasonicator (Q700, Qsonica (CT, USA)) at a power of 700 W, an amplitude of 80%, and a duty cycle of 50% for 6 h to prepare GnF samples. Next, the GnF samples were treated with a gamma-ray irradiator (Nordion, Canada) in which two gamma-rays with energy levels of 1.17 MeV and 1.34 MeV were produced by Co-60. Three doses (i.e., 1.0, 2.5, and 5.0 kGy) were accumulated at a dose rate of 10 kGy/h.

### Measurement and characterization

**Figure 1:**
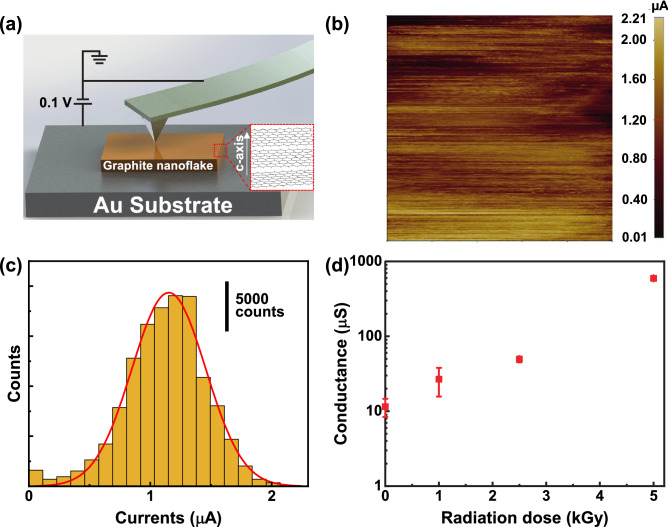
Electrical conductance of GnFs exposed to different gamma-ray radiation doses. (**a**) The schematic of CP-AFM for measuring electrical conductance. The inset in (**a**) shows the schematic of GnFs orientation in the measurement, the basal plane of GnFs parallel to the Au substrate. (**b**) 2D current map of GnF sample with no gamma-ray irradiation (i.e., 0.0 kGy). The scanning size is 250 × 250 nm^2^. (**c**) Current histogram obtained from the 0.0 kGy GnF. The current histogram is constructed from 2D current map [shown in (**b**)]. The value of average current is obtained by performing a curve fitting analysis to the current histogram with Gaussian distribution. (**d**) Electrical conductance versus gamma-ray radiation dose. An increase in gamma-ray radiation dose leads to an exponential increase in the electrical conductance of GnFs.

The c-axis electrical conductance of GnF samples was measured with CP-AFM in the commercial AFM system (NX-10, Park Systems (Korea)), as CP-AFM has been widely used to characterize the electrical properties of various nanomaterials such as molecule junction^18^, protein^19^, nanowires^20^, and thin film^21^. The GnF samples exposed to different gamma radiation doses were placed on Au substrate (Fig. 1a). Then, a platinum-coated probe tip with force constant of 2.8 N/m (NSC 18/Pt, MikroMasch (Estonia)) was in contact with the GnF samples at a constant force of 25 nN. To measure current, a DC voltage of 0.1 V was applied to the Au substrate and ground was applied to the GnF samples. It is worth noting that we first examined I-V curves to ensure that the CP-AFM measurements are in linear ohmic regime (see Fig. S1 in supplementary material). The internal structures of GnFs were characterized by Raman and XRD spectroscopy. All Raman measurements of GnF samples were performed with the DXR2xi Raman imaging microscope (Thermo Scientific (MA, USA)) using 532 nm laser radiation at room temperature. XRD spectra were collected on a Bruker D8 Advance diffractometer (MA, USA) with CuKα source ($\lambda =1.54060$ Å) operating at 40 mA and 40 kV in a parafocusing Bragg–Brentano mode. The measurement was performed with a step size of 0.02^0^ and 4 s per step. The morphology of GnFs was characterized by SEM and TEM. For SEM, GnFs were mounted with conductive carbon tape and viewed with a Hitachi S-4800 FESEM at an accelerating voltage of 5 kV. The TEM image was captured from a Hitachi HT7700 TEM at 100 kV and photographed with an AMT XR-41B 2 k × 2 k CCD camera.

## Results and discussion

The c-axis electrical conductance of the GnF samples exposed to different gamma radiation doses of 0.0, 1.0, 2.5, and 5.0 kGy was quantified with CP-AFM (Fig. 1a). For reference, the gamma radiation doses of 1.0, 2.5, and 5.0 kGy are approximately equivalent to the accumulated gamma-ray emitted from dry storage canisters (for spent nuclear fuels) for 16, 40, and 80 years, respectively. Each GnF sample was scanned in a contact mode with a scan size of 250 × 250 nm^2^ to obtain its 2D current map. A representative 2D current map of pristine GnF (i.e., 0.0 kGy GnF) is shown in Fig. 1b (see Fig. S2 in supplementary material for 2D current maps of GnFs with 1.0, 2.5, and 5.0 kGy). After obtaining the current map, data analysis was performed to obtain the average current by fitting a Gaussian distribution to the current histogram, as shown in Fig. 1c. A similar statistical analysis was carried out for 1.0, 2.5, and 5.0 kGy GnFs (Fig. S2 in supplementary material). The conductance *G* was then calculated from $G=I/V$, where *I* is the average current and *V* is the applied voltage. Our measurements show that the GnF without irradiation exhibits the lowest conductance value (Fig. 1d). With the accumulation of radiation dose, the conductance of GnF exponentially increases and reaches the maximum at 5.0 kGy radiation dose. We note that electrical contact area varies depending on Young’s modulus of samples^22^, which can potentially result in experiment errors. Xu et al. investigated the change of mechanical properties of carbon fiber under gamma-ray irradiation^11^. Even though they reported that Young’s modulus of carbon fiber increases with an increase of radiation dose, the change in Young’s modulus is considerably small at a low dose. Thus, we neglected Young’s modulus changes due to gamma-ray irradiation, and assumed the identical electrical contact area.

**Figure 2:**
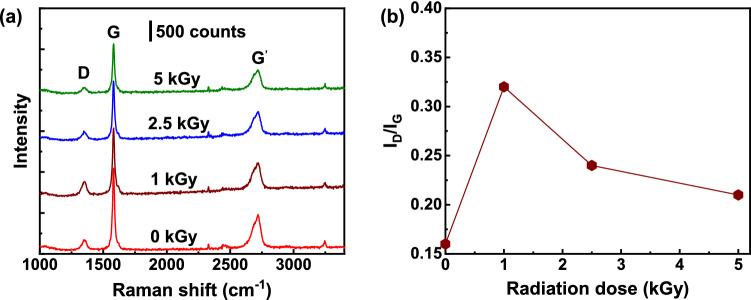
Raman spectroscopy analysis. (**a**) Raman spectra of GnF with varying gamma-ray radiation doses of 0.0, 1.0, 2.5, and 5.0 kGy. (**b**) I_D_/I_G_ ratio as a function of radiation dose.

To understand the change in the electrical properties of GnFs caused by gamma-ray irradiation, the internal structure of the GnFs was investigated with Raman spectroscopy and XRD measurement. Raman spectroscopy is a sensitive and non-destructive technique to investigate the degree of structural perturbation in carbon-based materials^23,24^. Figure 2a presents the Raman spectra of the GnF samples with radiation doses of 0.0, 1.0, 2.5, and 5.0 kGy. As clearly seen, two prominent peaks of graphite exist. A G-band peak (at about 1580 cm^−1^) represents in-plane vibrations of sp^2^ bonds, and a D-band peak (at about 1350 cm^−1^) represents the structural defect and disorder associated with sp^3^ hybridized carbon atoms. Another peak in the spectra is a G’-band (also called 2D band) positioned at about 2700 cm^−1^, which corresponds to the overtone of the D-band and represents intrinsic properties of well-ordered sp^2^ carbon atoms (without any kind of disorder)^25^. I_D_/I_G_ ratio is a useful parameter for evaluating the degree of graphitization of carbon-based materials. A higher I_D_/I_G_ ratio indicates a smaller degree of graphitization^26^. As the radiation dose increases, I_D_/I_G_ value first increases from 0.0 to 1.0 kGy, and then gradually decreases afterward (Fig. 2b). This observation indicates that the graphitization first decreases and moderately increases with the radiation dose. To understand the change in graphitization, it is worth considering the different mechanisms of interaction between gamma-ray irradiation and the GnFs.

There are three interactions between gamma-ray and solid materials: photoelectron, Compton scattering, and electron pair effect^27^. The influence of gamma-ray depends on the magnitude of gamma-quanta energy and the effective atomic number^12^. The photoelectric effect happens at low energy, whereas the electron pair effect is dominant in the high energy regime. At intermediate energy of around 1 MeV, the Compton scattering effect is the main mechanism of gamma-ray absorption. The effective atomic number of graphite can be calculated based on Mayneord equation^28^, ${Z}_{eff}={\left({a}_{1}{Z}_{1}^{m}+{a}_{2}{Z}_{2}^{m}+\dots +{a}_{n}{Z}_{n}^{m}\right)}^{1/m}$, where ${a}_{1},{a}_{2},\dots ,{a}_{n}$ are the fractional weight of compound elements, ${Z}_{1},{Z}_{2},\dots {,Z}_{n}$ are the elements atomic number, and $m$ is an exponent indicating power dependency. For practical purposes, $m$ is considered equal to be 2.94^29^. For the GnFs prepared by our method, the O:C atomic ratio is 0.042 as characterized in the previous study^30^. Then, the weight percentage of carbon and oxygen is 94.7% and 5.3%, respectively. Consequently, the effective atomic number of the GnFs can be calculated to be 6.14. The calculated effective atomic number and gamma-quanta energies of 1.17 MeV and 1.34 MeV indicate that the major interaction between gamma-ray and GnF is the Compton scattering effect^12^.

The interaction between Compton scattering effect and GnFs can be described as follows. The effect of gamma-quanta energy on defect creation and annealing involves an indirect process^31^. First, the incident photon interacts with carbon atoms of graphite to produce recoil electrons and scattered photons. Then, the recoil electrons collide with graphite atomic lattice and transfer energy. When the transferred energy exceeds an atomic displacement threshold energy, it generates vacancies through sputtering^32^. This threshold energy of so-called knock-on damage for single-walled carbon nanotubes is roughly 86 keV^33^. Gamma quanta energies used in this study are 1.17 and 1.34 MeV, which are likely sufficient to displace carbon atoms. Subsequently, the displaced atoms collide with other atoms, resulting in a collision cascade. Therefore, a large number of defects (i.e., displaced atoms) are generated and thereby disorder in structure increases. When sufficient defects are created to reach saturated level, transferred energy that is less than knock-on damage threshold energy is converted into thermal energy during the atomic collision process. As a result, local temperature increases, causing an annealing process. In this thermal annealing process, interstitial atoms diffuse and fill vacancy defects, and then crystal imperfections consequently are reduced.

Initial defect formation and subsequent crystal restoration upon gramma-ray irradiation are observed in this study. As seen in Fig. 2b, I_D_/I_G_ ratio increases from 0.0 to 1.0 kGy, indicating defect formation in GnF. Further increasing gamma-ray irradiation results in decreasing I_D_/I_G_ ratio, which can be attributed to the crystal restoration. We note, however, that I_D_/I_G_ ratio of GnF with 5.0 kGy is still higher than that of GnF with 0.0 kGy, suggesting that defects are not fully restored to the level of GnF without gamma-ray irradiation exposure. Our results are consistent with previous studies^13,32^. Cataldo used gamma-ray irradiation dose of 1 MGy with a dose rate of 5.7 kGy/h on graphite and found that the irradiated graphite presented more damage compared to the graphite without irradiation^13^. Wang et al. studied the effect of gamma-ray irradiation ranging from 0 to 3 MGy on graphene with a dose rate of 2.4 kGy/h^32^. They also suggested that the gamma-ray irradiation damaged graphene when the radiation dose increased from 0 to about 450 kGy, but the damage was gradually restored when the radiation dose further increased from 450 kGy to 3 MGy. They also found that the damage in graphene at 3 MGy was still greater than that in the pristine graphene. However, a study conducted by Li et al. shows results that are contradiction to those from Wang et al.’s work^12^. Li et al. used gamma-ray radiation doses of 200 kGy and 2 MGy on graphite with the dose rate of 1.8 kGy/h. They found that the degree of graphitization at 200 kGy was higher than that at 2 MGy, indicating no damage restoration in higher gamma irradiation dose. This discrepancy could be caused by the dose rate differences. Wang et al. used a higher dose rate of 2.4 kGy/h. In this study, we used even higher dose rate of 10 kGy/h, and our results are in agreement with those from Wang et al.’s work. A higher dose rate generates recoil electrons more rapidly. Thus, we suggest that even at a low radiation dose (1.0 kGy), but under a high dose rate, the amount of generated recoil electron is large enough to damage the graphite, while a further increase in the radiation dose leads to damage restoration.

**Figure 3:**
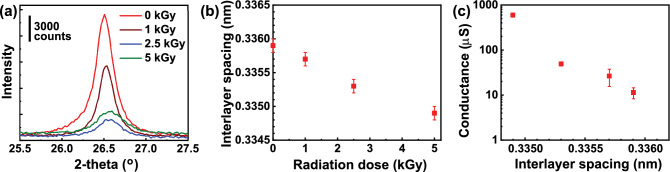
XRD spectroscopy analysis. (**a**) (002) peak of XRD spectra of GnF samples with varying gamma-ray radiation doses of 0.0, 1.0, 2.5, and 5.0 kGy. (**b**) Interlayer spacing of GnFs as a function of radiation dose. (**c**) Semi-log plot of electrical conductance as a function of interlayer spacing for GnFs. Exponential increase of conductance is observed as interlayer spacing is reduced.

While Raman spectroscopy revealed the degree of structure disorder, our findings from Raman spectroscopy cannot explain the conductance change upon gamma-ray irradiation, since no direct correlations were observed. In order to further elucidate the structure–property relationship, we performed XRD characterization. In XRD characterization, adding internal reference material to the sample helps obtain accurate results, since a minor error can result from the height difference of sample^34^. In this work, NiO was chosen as an internal reference material. The powder patterns were corrected based on the NiO referenced pattern (PDF 000–047-1049) from PDF 4 + database^35^. The enlarged XRD spectra of a peak (002) at $2\theta \approx {26.5}^{o}$ of our GnF samples with varying gamma-ray irradiation doses is shown in Fig. 3a. The full XRD spectra of GnFs and NiO can be found in Fig. S3 of the supplementary document. A closer look at the (002) peak reveals that it shifts to a higher angle with the increase of radiation dose. Based on the (002) peak position, interlayer spacing between the layers of GnFs can be calculated from the Bragg equation: $d=\frac{\lambda }{2\mathrm{sin}\theta }$ , where *d* is interlayer spacing, $\lambda$ is incident wavelength, and $\theta$ is the corrected Bragg angle. As the radiation dose increases from 0.0 to 5.0 kGy, the interlayer spacing of GnF monotonically decreases from 0.3359 to 0.3349 nm, as shown in Fig. 3b. Previous studies investigated the effect of gamma-ray irradiation with the range of 0 to 2 MGy on graphite^12,36^. Their results also showed that graphite’s interlayer spacing decreases as radiation dose increases. The values of 2$\theta$, interlayer spacing, and electrical conductance of gamma-ray irradiated GnFs are tabulated in Table S1 of supplementary material along with uncertainties of 2$\theta$ and interlayer spacing obtained from the peak fit using TOPAS5. We note that GnFs investigated in this work show interlayer spacing values very close to the value of pure graphite (0.3354 nm)^37^, indicating that graphite intrinsic structure is maintained.

The c-axis electrical conductance as a function of interlayer spacing for the gamma-ray irradiated GnFs is reported in Fig. 3c. It can be clearly seen that the conductance exponentially increases with the decrease of interlayer spacing, suggesting that the increase in electrical conductance of GnFs can be attributed to the reduced interlayer spacing. Prior studies have demonstrated a relation between interlayer spacing and electrical properties of graphite, which can support our experimental observations. Sutter et al. measured the c-axis electrical resistance of two-layer graphene while the separation between two layers was varied through different compressions with probes^38^. Their results show that electrical resistance exponentially decreases as the separation is reduced. In addition, Kozhemyakina et al. reported interlayer spacing dependence on electrical conductivity of graphite pellets^39^. Several grades of graphite were prepared, and their results indicate that the electrical conductivity decreases as interlayer spacing decreases. While these studies are relevant to our study, we note that more study is essential to elucidate a detailed mechanism behind the exponential dependence of electrical resistance on the interlayer spacing of gamma-ray irradiated GnFs. Several tunneling mechanisms can play a role in cross-plane charge transport of van der Waals layered materials. For example, Najmaei et al. employed a direct tunneling, Poole–Frenkel trap-based tunneling, and hopping conduction through the layers to describe out of plane charge transport in NbSe_2_ and HfS_2_^40^. While direct tunneling depends only on the applied electric field, trap-based tunneling and hopping conduction depend on temperature in addition to the electric field. Thus, temperature dependent current–voltage measurements on GnFs can enable quantitative analysis using the barrier models to discover which transport mechanisms are dominant in gamma ray irradiated GnF samples. Further, other factors such as stacking-faults and stacking sequence (i.e., Bernal vs. rhombohedral stacking) may also play a substantial role in c-axis transport, as it starts getting demonstrated in recent studies^41^. We also note that although I_D_/I_G_ ratio from Raman spectroscopy does not show a direct relation with c-axis conductance, defect density and types may play a role in c-axis charge transport, which warrants further study.

**Figure 4:**
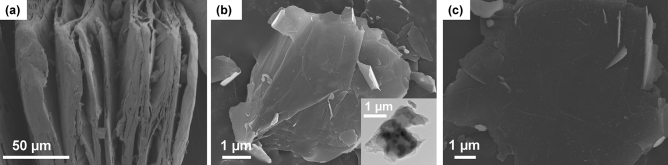
Characterization of GnF morphologies. (**a**) Intercalated graphite (before exfoliation). (**b**–**c**) GnFs with a gamma-ray radiation dose of (**b**) 0.0 kGy and (**c**) 5.0 kGy. Inset in (**b**) shows the TEM image.

Next, we checked the morphology of GnFs to examine the alterations resulting from the gamma-ray exposure. SEM and TEM images were obtained from intercalated graphite, GnF without gamma-ray exposure, and GnF after 5.0 kGy gamma-ray exposure (Fig. 4). For the intercalated graphite, the layered structure is clearly visible, as shown in Fig. 4a. SEM image is obtained for GnFs without gamma-ray irradiation (Fig. 4b). Figure 4c presents GnFs after 5.0 kGy gamma-ray irradiation. A comparison of the surface morphology between GnFs without gamma-ray irradiation and after 5.0 kGy radiation exposure reveals indistinguishable changes, suggesting that the gamma-ray irradiation up to 5.0 kGy does not induce notable changes on GnF surface. However, we note that the morphology of carbon-based materials could be changed if a higher radiation dose (order of 100 kGy) is applied as demonstrated in the previous work^42^.

## Conclusions

We enhanced the c-axis electrical conductance of small stacks of graphene (i.e., GnFs) through gamma-ray irradiation. The degree of enhancement in the electrical conductance was quantified with CP-AFM. Subsequently, internal structure and morphology (i.e., interlayer spsacing, graphitization, and morphology) were investigated with XRD, Raman spectroscopy, and SEM/TEM to find a relation between electrical property and structures. Change in low gamma radiation doses from 0.0 to 5.0 kGy leads to an exponential increase in the electrical conductance of GnFs. An increase in gamma-ray radiation results in a monotonic decrease in the interlayer spacing of GnFs, but their morphology change is unnoticeable. The experimental characterization suggests that the enhancement of c-axis electrical conductance of GnF might be attributed to a reduction of the interlayer spacing. Raman spectroscopy measurements indicate that knock-on damage occurs at 1.0 kGy, and graphitization is somewhat restored from 2.5 to 5.0 kGy, but not the extent of pristine GnFs (i.e., GnFs with no gamma-ray exposure). Our findings provide valuable insight into changes in the electrical properties and internal structures of GnFs under low gamma-ray radiation doses. In addition, this work will have significant implications for tailoring material properties of GnFs and other carbon-based materials.

## Supplementary Information

Supplementary Information.

## Data Availability

The data presented in this study are available on request from the corresponding authors.
