# Supplementary material for: Enhancing the electrical properties of graphite nanoflake through gamma-ray irradiation
Source: Sci Rep. 2022 Sep 1;12:14824. doi: 10.1038/s41598-022-19232-2 (PMC9437066; doi:10.1038/s41598-022-19232-2)
Supplement: Supplementary file 1 — Supplementary Information. [file 41598_2022_19232_MOESM1_ESM.docx]

**Supporting information**

for

**Enhancing the electrical properties of graphite nanoflake through gamma-ray irradiation**

*Anh Tuan Nguyen^1^, Youlim Lee^2^,* *Phuong Quang Hoang Nguyen^3^, Przemyslaw Dera^3^,
Sang-Hee Yoon^2,*^, Woochul Lee^1,*^*

*^1^Department of Mechanical Engineering, University of Hawaii at Manoa, Honolulu, Hawaii 96822, USA*

*^2^Bioinspired Engineering Laboratory, Department of Mechanical Engineering, Inha University, Incheon 22212, Republic of Korea*

*^3^Hawaii Institute of Geophysics and Planetology, University of Hawaii at Manoa, Honolulu, Hawaii 96822, USA*

**Correspondence to be addressed to, email: shyoon@inha.ac.kr, woochull@hawaii.edu*


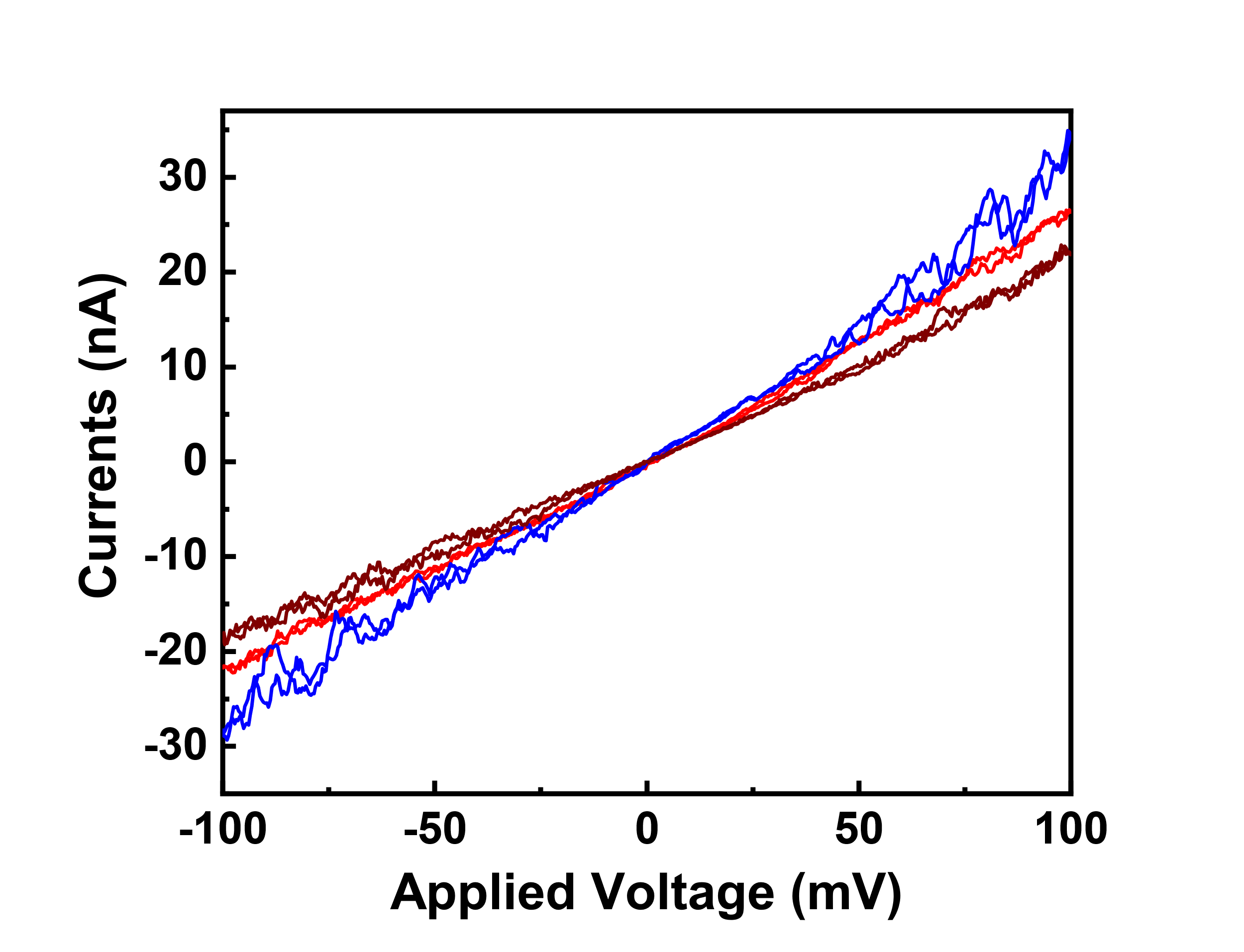


Figure S1: I-V curves characteristic of 0.0 kGy expandable graphite sample. Three colors represent I-V curves on three different locations. To determine a voltage for conductance measurements, we first conducted I-V curve measurement on the expandable graphite sample (we note that the expandable graphite is a bigger stack of graphene layers compared to graphite nanoflakes). Within the applied voltage window between –0.1V and 0.1V, I-V curves are linear. Therefore, we chose 0.1V to obtain the conductance map and histogram of graphite nanoflakes.

Table S1. List of 2*θ*, interlayer spacing, and electrical conductance of GnFs with varying gamma-ray radiation doses of 0.0, 1.0, 2.5, and 5.0 kGy. The values of 2*θ* and interlayer spacing are obtained from XRD measurement, and those of electrical conductance are gained from CP-AFM measurement.

| **Gamma radiation dose (kGy)** | $\boldsymbol{2}\boldsymbol{\theta(^{\circ})}$ | ***d_002_* (nm)** | **Electrical conductance (µS)** |
| --- | --- | --- | --- |
| 0.0 kGy | 26.515 $\pm$ 0.005 | 0.3359$\pm$ 0.0001 | 11.50 |
| 1.0 kGy | 26.530$\pm$ 0.003 | 0.3357 $\pm$ 0.0001 | 26.78 |
| 2.5 kGy | 26.564 $\pm$ 0.004 | 0.3353 $\pm$ 0.0001 | 49.40 |
| 5.0 kGy | 26.590$\pm$ 0.004 | 0.3349 $\pm$ 0.0001 | 593.60 |


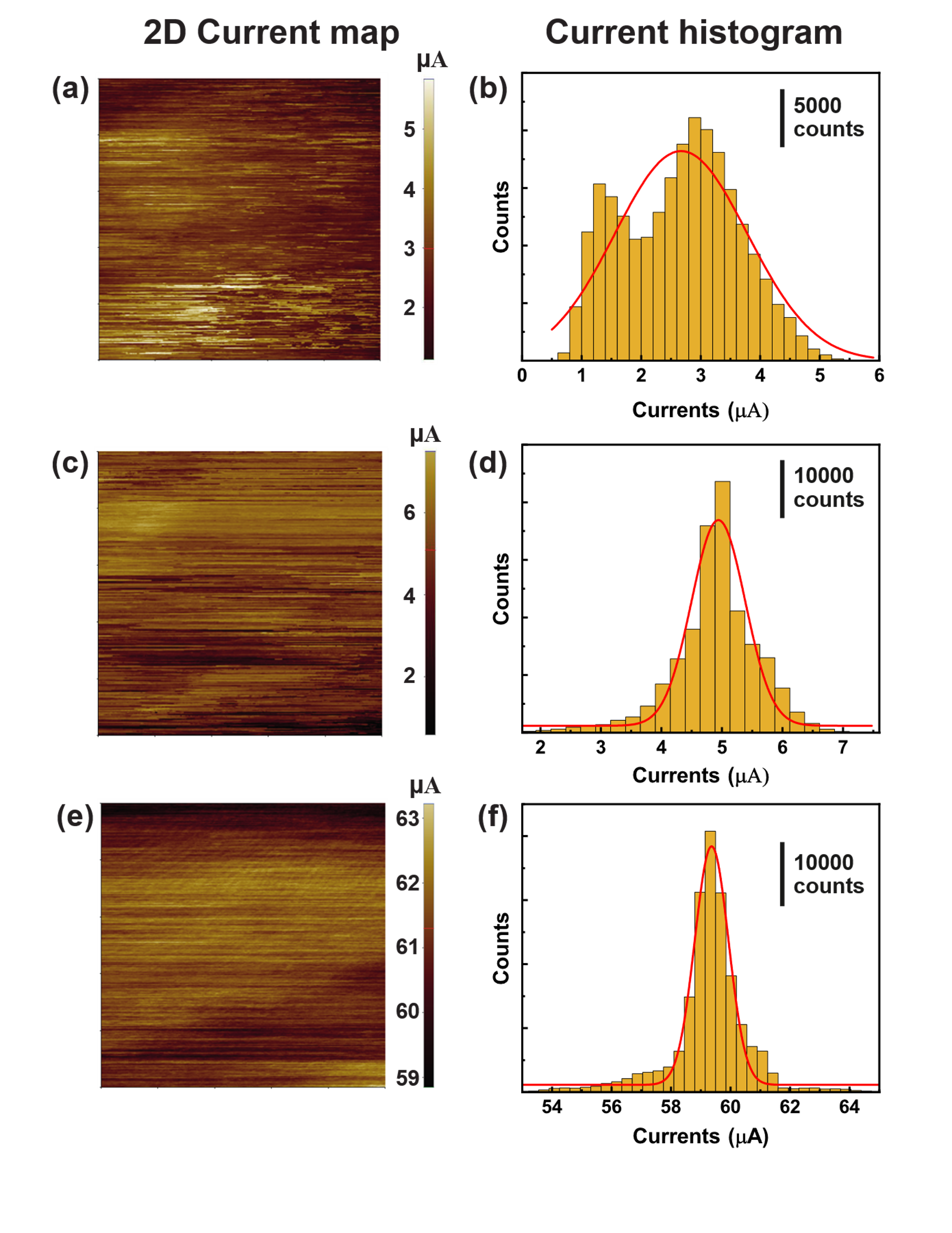


Figure S2. CP-AFM measurement of gamma-ray irradiated GnFs. 2D current map and current histogram are obtained from the GnFs with gamma-ray radiation doses of 1.0 kGy (a, b), 2.5 kGy (c, d), and 5.0 kGy (e, f). The size of current maps is 250 nm × 250 nm.


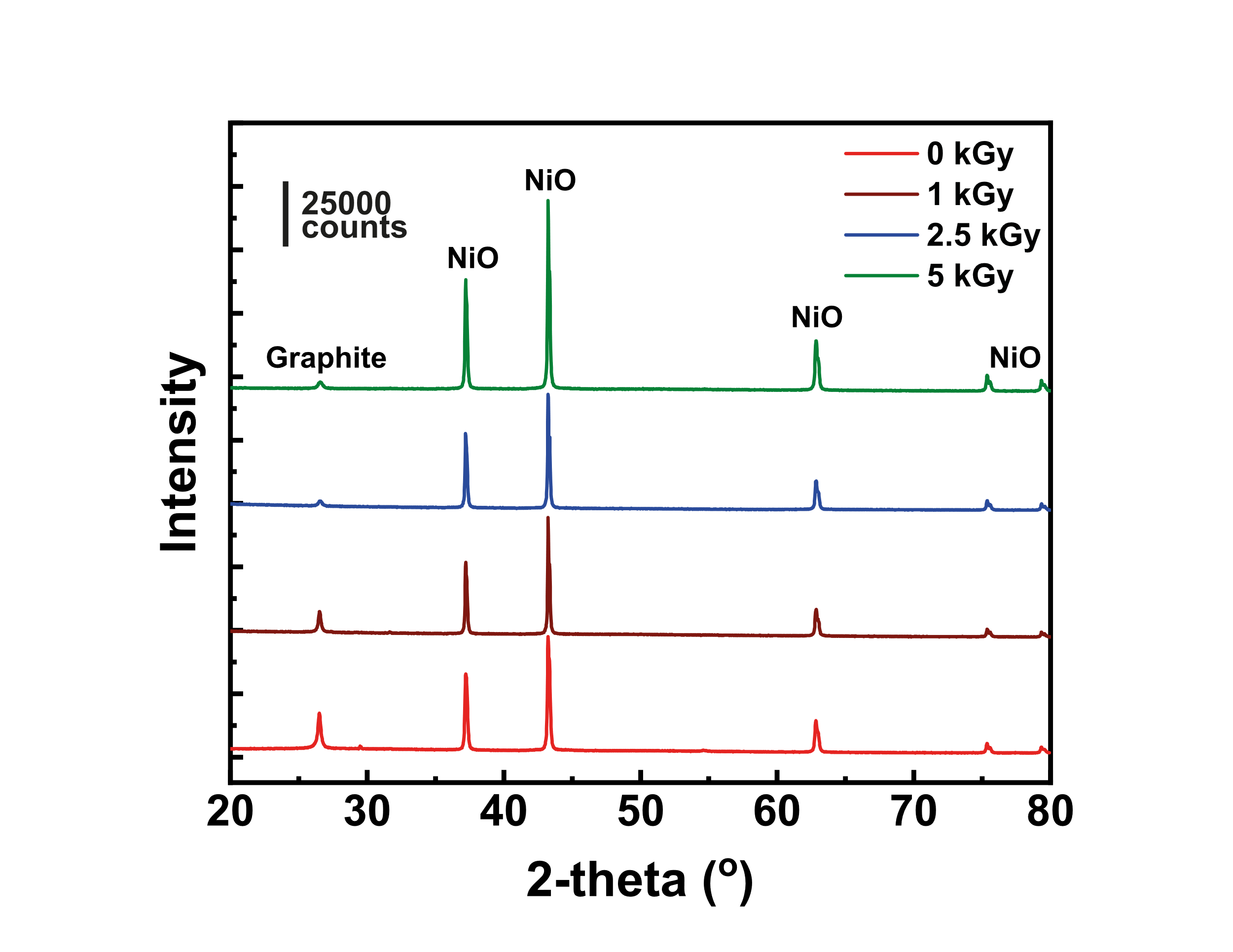


Figure S3. XRD spectra acquired from the GnFs with varying gamma-ray radiation doses of 0.0, 1.0, 2.5, and 5.0 kGy and NiO as internal reference material.
